# A clinicopathological features and treatment of lacrimal gland tumors at a tertiary eye hospital in Nepal

**DOI:** 10.1097/MS9.0000000000003113

**Published:** 2025-03-27

**Authors:** Jamuna Gurung, Rohit Saiju, Vera M. Beyuo, Dikshya Bista, Hom Bahadur Gurung, Purnima Rajkarnikar Sthapit

**Affiliations:** a Department of Ophthalmology, Pokhara Academy of Health Sciences, Pokhara, Nepal; b Department of Oculoplasty and Ocular Oncology, Tilganga Institute of Ophthalmology, Nepal; c Department of Ophthalmology, Korle Bu Teaching Hospital, Accra, Ghana

**Keywords:** histopathology, lacrimal gland tumor, treatment

## Abstract

**Background:**

To evaluate the clinico-demographic profile, histopathological findings, and treatment of patients with lacrimal gland tumors.

**Methods:**

This was a retrospective study of patients with lacrimal gland tumors diagnosed and managed at the Oculoplastic and Ocular Oncology department of a tertiary eye hospital in Nepal. Twenty-three patients with biopsy-proven lacrimal gland tumors between January 2017 and December 2021 were included. The clinical characteristics, histopathologic diagnosis, and their management were evaluated.

**Results:**

A total of 24 eyes of 23 patients were retrospectively reviewed. The mean age of patients was 41.67 ± 13.7 (17–60 years). Females 13 (54.2%) were more commonly affected. Most patients presented with a palpable mass in the periorbital region 10 (41.6%) followed by protrusion of the eyeball six (25%). The mean duration of symptoms was 10.8 months. The diagnosis was made clinically and radiologically and confirmed by histopathology. Surgical excision of the tumor was done in 18 (75%), followed by incision biopsy three (12.5%) and exenteration two (8.3%). Histopathology showed pleomorphic adenoma 11 (45.8%) as the most common lacrimal gland tumor, followed by adenoid cystic carcinoma seven (29.1%), adenocarcinoma three (12.8%), lymphoma two (8.3%) and carcinoma ex pleomorphic adenoma one (4.2%). Malignant tumors were treated by wide surgical excision followed by adjuvant treatment after consultation with an oncologist.

**Conclusion:**

The most common benign lacrimal gland tumor was pleomorphic adenoma and malignant was adenoid cystic carcinoma. The histopathological diagnosis was important to plan for the management of lacrimal gland tumors which have a significant role in the survival of the patient.

## Introduction

Tumors of the lacrimal gland constitute between 5% and 25% of all orbital tumors^[1-3]^. Lacrimal gland lesions comprise a wide spectrum of different entity from benign epithelial and lymphoid lesions to high-grade carcinomas, inflammatory lesions, and metastatic cancer. The most common epithelial tumors are, in order, pleomorphic adenoma, adenoid cystic carcinoma, and adenocarcinoma. Other rare variants of carcinoma include mucoepidermoid carcinoma, acinic cell carcinoma, and carcinoma ex pleomorphic adenoma^[4-6]^.

**Highlights**

- Tumors of the lacrimal gland constitute a wide spectrum of different entity from benign epithelial and lymphoid lesions to high-grade carcinomas, inflammatory lesions, and metastatic cancer.
- There is lack of data concerning lacrimal gland tumors and their management in Nepal.
- The histopathological diagnosis is important to plan for the management of lacrimal gland tumors which have a significant role in the survival of the patient.

A slow-growing painless mass, including displacement of the eyeball, decreased motility, diplopia, and ptosis characterize the benign lacrimal gland lesion. The rapid onset of symptoms associated with pain and radiologically bony destruction, calcification, and invasion in adjacent structures suggests malignant transformation. The diagnosis depends on the histopathological findings which helps in the choice of treatment and the prognosis. Surgical excision is the treatment for benign epithelial tumors, whereas carcinomas often require adjuvant radiotherapy and/or chemotherapy besides excision^[4]^.

Lacrimal gland tumors are a rare condition, few cases have been reported in Nepal^[7,8]^. So, we conducted this study in our attempt to find out the detailed clinico-demographic profile, histopathological types, and treatment of lacrimal gland tumors that have been presented to the Tilganga Institute of Ophthalmology.

## Methods

A retrospective review of 24 eyes of 23 patients diagnosed with lacrimal gland tumors managed during the 5 years from 1 January 2017 to 31 December 2021 done at the Oculoplastic and Ocular Oncology department of a tertiary eye hospital in Nepal. This was a retrospective study, with case series of patients with lacrimal gland tumors. The patient’s chief complaints, onset, duration, progression, pain, and other associated symptoms were taken from electronic medical records (EMR).

Visual acuity, extraocular movement (EOM), examination of the periorbital region, and proptosis were recorded. Anterior segment and dilated fundus evaluation were also noted. Data of the computed tomography (CT) and magnetic resonance imaging (MRI) of the head and orbit were retrieved. The diagnosis was made after orbital biopsy and histopathologic examination but in a few cases, the diagnosis was reached based on clinical and radiological findings.

The Institutional Review Committee approved the study TIO-IRC (Ref. 08/2022). The work was fully compliant with the STROCSS criteria^[9]^.

### Data analysis

Statistical Package for the Social Sciences 20.0 program analyzed and computed the data. Descriptive statistics like frequency, percentage, mean and standard deviation were used to describe the characteristics of collected data.

## Results

The study included 24 eyes with a mean age of 41.67 ± 13.7 (range, 17–60) years. 13 (54.2%) were females and 11 (45.8%) were males. The left eye 12 (50%) was affected more than the right eye 11 (45.8%). The patients presented with a palpable mass in the periorbital region 10 (41.6%), protrusion of the eyeball six (25%), followed by ocular and periorbital pain three (12.5%), and diminution of vision three (12.5%) (Table 1). The mean duration of symptom presentation was 10.8 months.

**Table 1:** Patients clinico-demography

Parameters	Number	Percentage
Age (years)		
≤20	1	4.2
21–40	8	33.3
>40	15	62.5
Sex		
Female	13	54.2
Male	11	45.8
Laterality		
Left	12	50
Right	11	45.8
Both	1	4.2
Anatomical location		
Lateral	10	41.6
Superior	8	33.3
Supero-lateral	6	25
Clinical presentation		
Periorbital mass	10	1.6
Protusion of eyeball	6	25
Protusion of eyeball	6	25
Diminution of vision	3	12.5
Double vision	2	8.3
Restricted ocular movement	1	4.2

**Figure 1.**
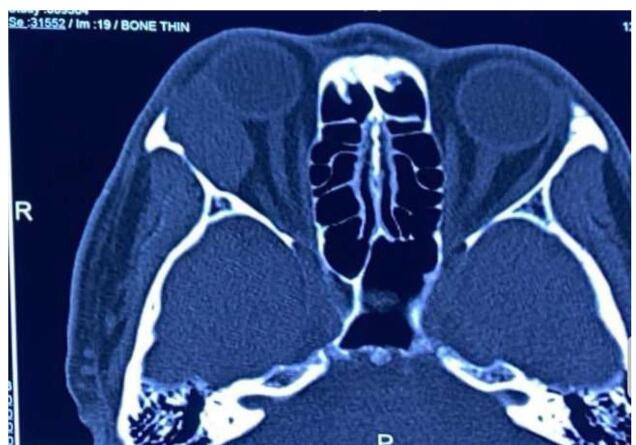
Axial view CT scan showing an ill-defined lobulated mass in the right lacrimal fossa with infiltration into the lateral bony wall, case of adenoid cystic carcinoma.

Besides clinical diagnosis, imaging like CT scans and MRI was used to find the anatomical location, consistency, bony involvement, and metastasis of tumors. A case presented with a palpable mass in the forehead and zygomatic region, of which CT showed a diffuse mass in the left lacrimal gland with bony erosion of lateral and superior orbital wall with an extension of mass to the brain parenchyma and zygomatic fossa which was suspected to be adenoid cystic carcinoma (Fig. 1).

**Figure 2.**
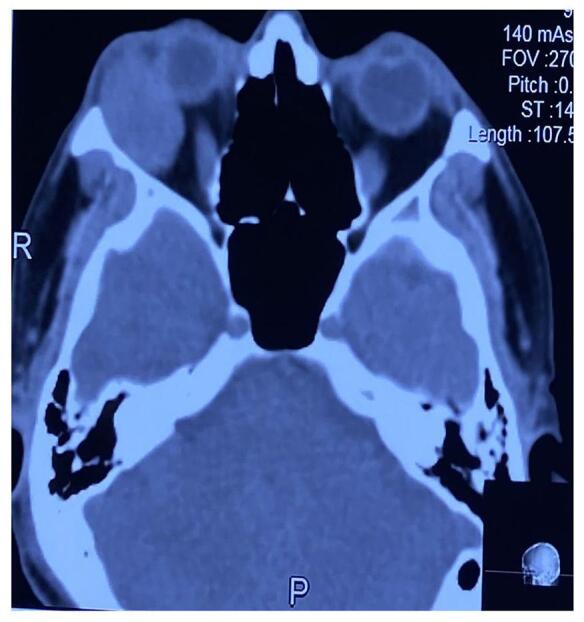
A CT scan shows a well-defined lobulated round, soft tissue density space-occupying lesion in the lacrimal gland fossa with proptosis in the right eye.

Orbitotomy with en-bloc resection of the tumor with an intact capsule was performed in patients with suspected pleomorphic adenoma (Fig. 2). Lateral orbitotomy with resection of tumors was performed in seven (29.2%) and three (12.5%) patients who underwent incisional biopsy. Two (8.3%) cases required orbital exenteration due to diffuse orbital involvement, including extraocular muscles (Table 2).

**Table 2:** Types of surgical procedures

Type of surgical management	Number of eyes (%)
Excision of tumor	18 (75.0)
Incisional biopsy	3 (12.5)
Exenteration	2 (8.3)
Total	24

**Figure 3.**
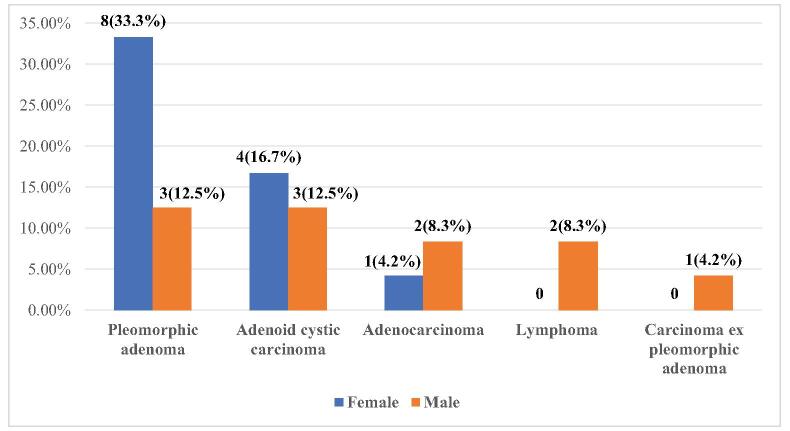
Types of lacrimal gland tumors.

Histopathology showed pleomorphic adenoma in 11 (45.8%) followed by adenoid cystic carcinoma in seven (29.1%) (Fig. 3). The presentation and diagnosis of carcinoma ex pleomorphic adenoma were earlier (5 months) compared to pleomorphic adenoma (12 months) and other lacrimal gland tumors.

**Figure 4.**
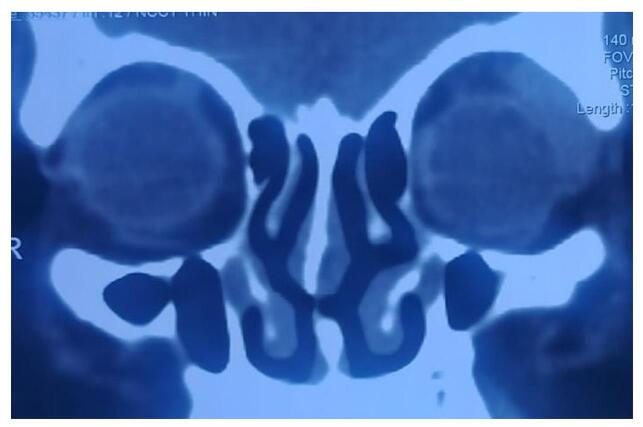
A coronal CT showing diffusely enlarged lacrimal gland in a 56-year-old man in the left eye suggestive of lymphoma.

After histopathological diagnosis, metastatic work-up was done for all the malignant cases. These patients were referred to an oncologist for external beam radiotherapy. Chemotherapy was given to a patient with lacrimal gland lymphoma (Fig. 4).

Pleomorphic adenoma has been followed up for an average of 26.2 months, lymphoma 7.5 months, and carcinoma ex pleomorphic adenoma 36 months, showing no recurrences. A case of adenoid cystic carcinoma (ACC) showed metastasis into intracranial and regional lymph nodes at the time of diagnosis who died three years after the diagnosis.

Another case of adenocarcinoma showed lymph node metastasis (Table 3). These patients were referred to an oncologist for further management.

**Table 3:** Clinical characteristics of lacrimal gland tumors

Histopathological diagnosis	Time to presentation (average months)	Modes of treatment	Average time follow-up (months)	Metastasis (number)
Pleomorphic adenoma	12	Surgery	26.2	0
Adenoid cystic carcinoma	6.8	Surgery and Radiotherapy	21.6	Brain (1), Lymph node (1)
Adenocarcinoma	5.5	Surgery and Radiotherapy	12	Lymph node (1)
Lymphoma	7.5	Surgery, Radiotherapy and Chemotherapy	7.5	0
Carcinoma ex pleomorphic adenoma	5	Surgery and Radiotherapy	36	0

## Discussion

Our study showed the mean age of diagnosis 41.67 ± 13.7 years, similar to another study (41.3 years)^[10]^. Lacrimal gland tumors usually affect the middle-aged population and are rare in children and adolescents with only a few reported cases similarly observed in our study^[10,11]^. Von Holstein *et al* reported benign tumors were significantly found more in younger age than malignant tumors (48 versus 63 years, *P* < 0.001)^[1]^. In studies, malignant tumors were seen later in life, after 5th decade^[1,12]^.

Although there was a female predominance in our study, a greater number of males presented with malignant tumors similar to another study^[10]^. Other studies showed no gender preference^[13,14]^.

In our study, benign tumors most commonly presented as a painless palpable mass of slow growth and eyeball protrusion, similar to other literature^[14,15]^. Malignant lacrimal gland tumors presented with a short history (<12 months) of painful palpable mass with rapid growth, displacement of the globe, and reduced visual acuity. Pain can be probably due to perineural infiltration or bone involvement in an advanced stage of malignant tumors^[14]^.

Shield et al, reported 55% benign epithelial lacrimal gland tumors and 45% malignant tumors^[16]^. Our study found more malignant tumors (54.2%) than benign tumors. Our hospital is a tertiary eye care center with an oncology clinic, so advanced cases are being referred here for further management, which can be a cause of the increased number of malignant cases in our research.

Adenoid cystic carcinoma was the most frequent malignant tumor of the lacrimal gland similar to previous findings^[14,17]^. This was followed by adenocarcinoma of the lacrimal gland. A study from Nepal reported lacrimal gland tumors in 14.9% of all orbital tumors of which adenoid cystic carcinoma 4.3% and lymphoma 4.3% were common pathological diagnoses^[7]^. Another study reported 2.56% adenoid cystic carcinoma among all the orbital tumors (11.11%)^[8]^.

Radio-imaging (CT scan or MRI) was performed to look for the lesion’s anatomical location, tumor extension, and consistency. Lacrimal gland tumors were managed surgically by en-bloc excision of the tumor in benign lesions. In contrast, malignant tumors received a combination therapy of surgery, postoperative radiotherapy, and chemotherapy after histopathological diagnosis and consultation with an oncologist. Three (12.5%) patients underwent an incisional biopsy which showed adenocarcinoma, ACC, and lymphoma respectively. Exenteration was performed in two ACC cases. There has been a recurrence in one case of adenocarcinoma to date. Mortality was found in one case of adenoid cystic carcinoma due to CNS metastasis three years after the diagnosis.

Because of the retrospective nature of the study, we had to exclude patients with incomplete EMRs. So, further, prospective studies with a larger sample size should be conducted in the future.

## Conclusion

The most common benign lacrimal gland tumor was pleomorphic adenoma and malignant was adenoid cystic carcinoma, the majority of the cases presented after the fourth decade of life. Unlike benign tumors, malignant tumors had rapid growth with painful mass. History, clinical examination, and radiological diagnosis aid in diagnosis but histological examination helps in confirmative diagnosis. The histopathological diagnosis is important to plan for the management of lacrimal gland tumors which have a significant role in the survival of the patient.

The small sample size and retrospective nature are the limitations of the study.

## Registration of research studies

Research Registry (researchregistry10521).
